# Co-expression of TNF receptors 1 and 2 on melanomas facilitates soluble TNF-induced resistance to MAPK pathway inhibitors

**DOI:** 10.1186/s12967-022-03538-w

**Published:** 2022-07-25

**Authors:** Cindy A. Sander, Elizabeth A. Rush, Jian Shi, Lidia M. R. B. Arantes, Raymond J. Tesi, Mark A. Ross, Michael J. Calderon, Simon C. Watkins, John M. Kirkwood, Robert L. Ferris, Lisa H. Butterfield, Lazar Vujanovic

**Affiliations:** 1 UPMC Hillman Cancer Center, University of Pittsburgh, L2.19 Hillman Cancer Center, 5117 Centre Avenue, Pittsburgh, PA USA; 2 Department of Medicine, University of Pittsburgh, Pittsburgh, PA USA; 3 Department of Otolaryngology, University of Pittsburgh, Pittsburgh, PA USA; 4 Molecular Oncology Research Center, Barretos Cancer Hospital, Barretos, SP Brazil; 5 INmune Bio, La Jolla, CA USA; 6 Department of Cell Biology, University of Pittsburgh, Pittsburgh, PA USA; 7 Department of Immunology, University of Pittsburgh, Pittsburgh, PA USA; 8 School of Medicine Department of Surgery, University of Pittsburgh, Pittsburgh, PA USA; 9 Parker Institute for Cancer Immunotherapy, San Francisco, CA USA

**Keywords:** Melanoma, Soluble TNF, TNF receptor 1, TNF receptor 2, CD271, Drug resistance, BRAF, MEK, Inhibitors

## Abstract

**Background:**

The effectiveness of MAPK pathway inhibitors (MAPKi) used to treat patients with BRAF-mutant melanoma is limited by a range of resistance mechanisms, including soluble TNF (solTNF)-mediated NF-kB signaling. solTNF preferentially signals through type-1 TNF receptor (TNFR1), however, it can also bind to TNFR2, a receptor that is primarily expressed on leukocytes. Here, we investigate the TNFR2 expression pattern on human BRAF^V600E+^ melanomas and its role in solTNF-driven resistance reprogramming to MAPKi.

**Methods:**

Flow cytometry was used to test TNFR1, TNFR2 and CD271 expression on, as well as NF-kB phosphorylation in human BRAF-mutant melanoma. The ability of melanoma cell lines to acquire MAPKi resistance in response to recombinant or macrophage-derived TNF was evaluated using the MTT cytotoxicity assay. Gene editing was implemented to knock out or knock in TNF receptors in melanoma cell lines. Knockout and knock-in cell line variants were employed to assess the intrinsic roles of these receptors in TNF-induced resistance to MAPKi. Multicolor immunofluorescence microscopy was utilized to test TNFR2 expression by melanoma in patients receiving MAPKi therapy.

**Results:**

TNFR1 and TNFR2 are co-expressed at various levels on 4/7 BRAF^V600E+^ melanoma cell lines evaluated in this study. In vitro treatments with solTNF induce MAPKi resistance solely in TNFR2-expressing BRAF^V600E+^ melanoma cell lines. TNFR1 and TNFR2 knockout and knock-in studies indicate that solTNF-mediated MAPKi resistance in BRAF^V600E+^ melanomas is predicated on TNFR1 and TNFR2 co-expression, where TNFR1 is the central mediator of NF-kB signaling, while TNFR2 plays an auxiliary role. solTNF-mediated effects are transient and can be abrogated with biologics. Evaluation of patient specimens indicates that TNFR2 is expressed on 50% of primary BRAF^V600E+^ melanoma cells and that MAPKi therapy may lead to the enrichment of TNFR2-expressing tumor cells.

**Conclusions:**

Our data suggest that TNFR2 is essential to solTNF-induced MAPKi resistance and a possible biomarker to identify melanoma patients that can benefit from solTNF-targeting therapies.

**Supplementary Information:**

The online version contains supplementary material available at 10.1186/s12967-022-03538-w.

## Background

Melanoma is the most lethal form of cutaneous cancer, being responsible for 80% of skin cancer-related deaths [1]. Somatic mutations in BRAF, a key protein kinase in the mitogen-activated protein kinase (MAPK) signaling pathway, lead to oncogenic programming in pre-malignant melanocytes. Approximately 50% of melanomas harbor mutations at position 600 of the BRAF gene (V600), the majority of which involve the substitution of valine by glutamic acid (V600E), resulting in constitutively active BRAF [2]. Vemurafenib, dabrafenib (BRAF inhibitors; BRAFi) and trametinib (MAPK kinase inhibitor; MEKi) have been FDA-approved for the treatment of human cancers harboring BRAF^V600E^ mutation [3]. Their use is associated with prolonged survival and progression-free survival in a notable fraction of patients with previously untreated BRAF^V600E^ mutated melanoma and, in the adjuvant postoperative setting, there appears to be long-term durable relapse-free and survival benefit [4]. Unfortunately, most of the patients treated with BRAFi and/or MEKi exhibit disease progression due to the onset of resistance, which principally occurs via reactivation of the MAPK pathway [5–7].

The effectiveness of MAPK cascade-targeting inhibitors (MAPKi) has thus far been limited by a range of intrinsic, adaptive and acquired mechanisms mediating resistance to MAPKi [8]. Acquired resistance to MAPKi can be mediated by exogenous factors that originate from the tumor microenvironment (TME), particularly from tumor-associated macrophages (TAM). It has been shown that treatment with MAPKi leads to increased numbers of TAM in melanoma lesions, and that tumor necrosis factor (TNF) released by TAMs induces melanoma resistance to MAPKi, blocking apoptosis mediated by inhibition of BRAF signaling [9–11]. These studies indicate that selective inhibition of TNF-mediated signaling may enhance the therapeutic benefits associated with MAPKi.

TNF signaling is mediated through type-1 (TNFR1) and type-2 TNF receptors (TNFR2). TNFR1 and TNFR2 are preferentially activated by soluble (solTNF) and transmembrane TNF (tmTNF), respectively, and have structurally distinct intracellular domains that activate different signaling pathways [12]. TNFR1 generally mediates apoptosis, cell survival and cytokine secretion, while TNFR2 selectively mediates cell survival and cytokine secretion [12]. While TNFR1 is ubiquitously expressed on most cell types, TNFR2 expression is predominantly described on immune cells [12]. TNFR2 expression has been reported on melanoma cell lines [13], however, the prevalence and function of TNFR2 on melanomas (BRAF-mutated in particular) is poorly understood.

In the present study, we investigated the prevalence of TNFR2 expression by human BRAF^V600E+^ melanomas and whether it plays a role in TNF-mediated resistance reprogramming to MAPKi. We show that 50% of BRAF^V600E^-mutant melanomas express TNFR2, that co-expression of TNFR1 and TNFR2 on these tumors is required for solTNF-mediated induction of MAPKi resistance and that selective neutralization of solTNF can mitigate solTNF-mediated MAPKi resistance. Cumulatively, our data suggest that TNFR2 could be utilized as a biomarker to identify melanoma patients that may benefit from TNF-targeting therapies.

## Methods

### Cell lines

**Fig. 1:**
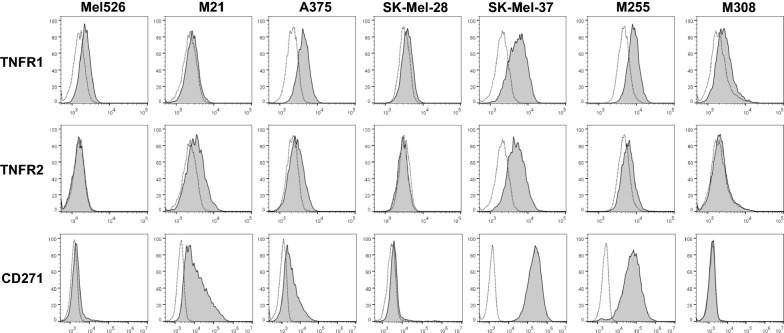
BRAF^V600E+^ melanoma cell lines display different expression patterns of TNFR1, TNFR2 and CD271. Seven human BRAF^V600E+^ melanoma cell lines were evaluated for TNFR1, TNFR2 and CD271 expression levels by flow cytometry. Cells were stained by two-step staining.  Dotted line–IgG control. Gray histogram–TNF receptor staining. BRAF mutation status was determined by PCR and flow cytometry

Mel526, M21, A375, SK-Mel-28, SK-Mel-37, M255 and M308 melanoma cell lines [14–16] were either obtained from ATCC or were kindly provided by Dr. John M. Kirkwood. Cells were phenotyped by flow cytometry (Fig. 1; Additional file 1: Table S1). They were cultured in complete cell-culture medium [RPMI 1640 medium, supplemented with 10% fetal bovine serum, 1% penicillin–streptomycin, 1% non-essential amino acids and 1% L-glutamine (Life Technologies; Carlsbad, CA)], at 37 °C, in a humidified 5% CO_2_ atmosphere, and were passaged bi-weekly. The cell lines were negative for mycoplasma contamination as shown by GEN-PROBE Mycoplasma Tissue Culture Non-Isotopic Rapid Detection System (Gen-Probe, Inc.; San Diego, CA).

### BRAF^V600E^ screening by PCR

DNA was extracted from melanoma cell lines using the DNeasy Tissue Kit (Qiagen) according to the manufacturer’s protocol. Extracted DNA samples were screened for BRAF mutation by PCR as previously described [17] using the following primer set forward: 5′-TCATAATGCTTGCTCTGATAGGA-3′; reverse: 5′- GGCCAAAAATTTAATCAGTGGA-3′. The PCR reaction parameters consisted of an initial 30 s denaturation step at 98 °C followed by 30 amplification cycles that consisted of denaturation at 98 °C for 30 s, annealing at 63 °C for 30 s, and extension at 72 °C for 30 s. The final cycle was followed by an additional extension step at 72 °C for 10 min. Data are summarized in Additional file 1: Table S1.

### Patient sample acquisition and storage

With the UPMC Hillman Cancer Center (HCC) IRB-based informed consent, tumor biopsies were obtained from non-trial melanoma patients (HCC #96-099), as well as melanoma patients enrolled in the trial that tested safety and efficacy of concurrent vemurafenib and high-dose interferon-α2b (IFN-α2b) therapy (HCC #12-107). Patient characteristics are described in Additional file 1: Table S2. Bulk melanoma single cell suspensions were collected and cryopreserved as previously reported [18]. All patient specimens were processed by competency-trained technologists under standard operating procedures in the Immunologic Monitoring Laboratory.

### Tumor cell line treatments

Recombinant human TNF (rhTNF; 12.5–100 ng/ml; R&D Systems; Minneapolis, MN) was used as a solTNF homologue. Dominant-negative TNF (DN-TNF; XPro1595/INB03; INmune Bio) is a next generation biologic that selectively neutralizes solTNF via quantitative exchange of individual subunits from the solTNF homotrimer [19]. For neutralization experiments, rhTNF was incubated in the presence of anti-human TNF antibody (R&D Systems; 10 µg/ml) or DN-TNF (10 µg/ml) in complete cell-culture medium at 37 °C for 1 h prior to cell treatment to ensure complete solTNF sequestration. Melanoma cell lines were cultured in 6-well plates (Corning, Manassas, VA) in the presence or absence of unscathed or pre-neutralized rhTNF for 48–72 h. Subsequently, melanomas were collected by gentle enzymatic dissociation using TrypLE Select (ThermoFisher Scientific; Pittsburgh, PA) and their phenotype and responsiveness to MAPKi were evaluated.

### Macrophage generation and activation

Peripheral blood mononuclear cells (PBMCs) were separated from healthy donor blood (collected in heparin anti-coagulant under the HCC #04–001 protocol) using Ficoll Hypaque gradient centrifugation (Corning) and CD14^+^ monocytes were isolated by magnetic sorting (Miltenyi Biotec; Auburn, CA) as previously described [20]. Monocytes were seeded in 6-well plate (24 mm-diameter) 0.4 μm pore polyester membrane inserts (Corning; Corning, NY) and cultured in AIM-V media supplemented with 2% HuAB serum (ThermoFisher Scientific) and 100 IU/ml macrophage colony-stimulating factor (M-CSF; PeproTech, Inc.; Rocky Hill, NJ). On day 5 of culture, macrophages were activated with 1000 IU/mL interferon-γ (IFN-γ; PeproTech) and 250 ng/mL lipopolysaccharide (LPS; Sigma-Aldrich) for 3 h. Prior to setting up transwell co-culture experiments, media was carefully suctioned out of inserts and cells were gently rinsed in wells containing PBS (Gibco; Waltham, MA).

### Transwell experiments

The experiments were performed in 6-well plates (Corning). Tumor lines were seeded at 2–5 × 10^5^ cells per 2.6 ml in the 24 mm wells. Transwell inserts containing activated macrophages (described in previous paragraph) were placed into melanoma-containing wells. After 48 h of co-culture in the presence or absence of anti-human TNF antibody (10 µg/ml) or DN-TNF (10 µg/ml), melanoma cells were collected using TrypLE Select (ThermoFisher), and their phenotype and MAPKi responsiveness were tested.

### Measurement of secreted cytokines

Activated macrophage-conditioned media was assessed by Luminex for released cytokines, including solTNF and vascular endothelial growth factor (VEGF) using the Cytokine Human 30-Plex Panel (ThermoFisher).

### CRISPR/Cas9 editing of melanoma cell lines

TNFR1, TNFR2 and CD271 (NGFR p75) gene knockout plasmids and transfection reagents (UltraCruz Transfection Reagent; Plasmid Transfection Medium; Santa Cruz Biotechnology; Dallas, TX) were used to genetically modify the SK-Mel-37 cell line per manufacturer’s instructions. TNFR2 knockin plasmid (R&D Systems) and transfection reagents (Lipofectamine 3000; Opti-MEM media; ThermoFisher) were used to generate the TNFR2-expressing SK-Mel-28 cell line per manufacturers' instructions. Stable transfectants were enriched by fluorescence activated cell sorting using the MoFlo Astrios High Speed Sorter (Beckman Coulter; Brea, CA).

### Flow cytometry

Melanoma cell lines were initially assessed by two-step staining for TNFR1, TNFR2 (R&D Systems; Minneapolis, MN) and CD271 (BD Biosciences; San Diego, CA) expression levels. Cells were stained with phycoerythrin (PE)–conjugated polyclonal goat anti–mouse immunoglobulin F(ab′)2 (Dako North America) as previously reported [21]. Matching IgG isotype controls were obtained from the same vendors. One-step staining of cell-surface antigens on melanoma biopsies and CRISPR/Cas9 edited cell lines was performed as previously reported [21, 22] using the following reagents: LIVE/DEAD™ Fixable Red Dead Cell Stain Kit (ThermoFisher); mouse anti-human CD45-Alexa Fluor 700 (clone HI30), CD90-PE-Cy7 (clone 5E10), CD271-BV510 (clone C40-1457; BD Biosciences), TNFR1-PE (clone 16803) and TNFR2-APC (clone 22235; R&D Systems) monoclonal antibodies, and matching IgG isotype controls from the same vendors. For intracellular staining, melanoma cell lines were fixed, permeabilized and stained using the FoxP3 Staining Buffer Set (Miltenyi Biotec; Auburn, CA) per manufacturer’s protocol. Cells were stained for anti-NF-kB p65 (pS529)-PE (clone K10-895.12.50; BD Biosciences), anti-p38 MAPK (pT180/pY182) Alexa Fluor 647 (clone 36/p38; BD Biosciences) or BRAF^V600E^ [(clone VE1; counterstained with anti-mouse Alexa Fluor 488 (Abcam)]. Flow cytometry analyses were performed using BD Accuri C6 and BD LSRFortessa™ cell analyzers, and evaluated using FlowJo v10 (FlowJo, LLC; Ashland, OR) software.

### 3-(4,5-dimethylthiazol-2-yl)-2,5-diphenyltetrazolium bromide (MTT) cytotoxicity assay

Following their stimulation with TNF ± TNF inhibitors, melanoma cell lines were collected and seeded at 0.5–1.5 × 10^4^ cells/well in flat-bottom 96-well plates (Corning). Sensitivity of melanoma cell lines to BRAFi (PLX4720) and MEKi (selumetinib; Selleckhem; Houston, TX) was assessed against pre-determined E.D._50_ concentrations for each inhibitor. The total media/inhibitor volume per well was 100 µl. Following a 48 h incubation, 10 µl of the MTT stock solution (5 mg/ml PBS; Sigma) was directly added into each cell-containing well. Following a subsequent 3 h incubation at 37 °C, media was completely removed and replaced with 150 µl of 99.9% isopropanol (Sigma) per well. To optimally solubilize formazan that was formed in viable cells, the plate was shaken for 30 min on a microplate shaker at room temperature. Absorbance of color intensity of DMSO-solubilized formazan was measured on the Epoch microplate spectrophotometer (BioTek; Winooski, VT) using the 570 nm emission wavelength. The percentage of cytotoxicity was calculated using the following formula: % killing = (E–S)/(TD − S) × 100, where E is experimental well, S is spontaneous death (untreated target cells) and TD is total cell death (target cells lysed with ddH_2_O).

### Immunofluorescence microscopy

Human melanoma OCT sections (5 μm) were fixed with 2% paraformaldehyde for 20 min. Sections were stained overnight with 2 µg/ml of Alexa Fluor 546 mouse anti-human TNFR2 (Santa Cruz; Ab clone D-2) and Alexa Fluor 647 rabbit anti- human SOX10 (Abcam; Ab clone SP267) antibodies. Nuclei were stained with Hoechst (Sigma) 1 mg/100 ml dH_2_0 for 1 min, washed in PBS, and mounted in Gelvatol (Sigma). Large area scan images at 40 × magnification were obtained on Nikon A1 confocal microscope with NIS Elements v5.2 (Nikon Instruments Inc; Melville, NY). To confirm forced TNFR2 expression on the SK-Mel-28 cell line, 3 × 10^5^ melanoma cells were seeded in 6-well plates (Corning) and cultured overnight at 37 °C. Cells were directly fixed in the wells using 2% paraformaldehyde for 30 min at 4 °C. Cells were stained for 1 h at room temperature with 10 µg/ml of anti-human TNFR2 antibody (clone #22221; R&D Systems) and were subsequently stained with Alexa Fluor 488–conjugated goat anti–mouse immunoglobulin F(ab′)2 (Cell Signaling Technology; Danvers, MA). Nuclei were labeled with 300 nM DAPI (ThermoFisher) for 10 min at room temperature. Images at 20X magnification were generated using the BioTek Lionheart FX automated microscope and Gen5 Image + v3.05 software (BioTek).

### Statistical analysis

When comparing two test groups, two-tailed Student’s *t*-tests were used for all analyses. When comparing three or more groups, Kruskal–Wallis test was used to get the overall p-value, followed by the Wilcoxon rank-sum to determine pairwise p-values. For correlation plots, statistical significance was estimated by linear correlation. To explore the relationship between a cell line’s sensitivity to MAPKi following solTNF treatments and its expression levels of markers (as measured by mean fluorescence intensity; MFI), the data were log2-transformed and Spearman’s correlation was used. Graphs were generated using GraphPad Prism v6 (GraphPad Software, Inc., La Jolla, CA). *P* values ≤ 0.05 were considered to be statistically significant.

## Results

### BRAF.^V600E+^ melanoma cell lines display different expression patterns of TNFR1, TNFR2 and CD271

BRAF^V600E^ mutation status of Mel526, SK-Mel-28, SK-Mel-37, M255, M308, M21 and A375 cell lines was confirmed by PCR (Additional file 1: Table S1). For each cell line, we determined the median effective dose (E.D._50_) for BRAFi and MEKi. The E.D._50_ for BRAFi ranged from 0.5 (A375) to 27 µM (M308), while E.D._50_ for MEKi ranged from 0.1 (Mel526) to 12 µM (M308; Additional file 1: Table S1). Cell-tailored MAPKi E.D._50_ doses were used in subsequent killing assays. Flow cytometric analysis indicated that genotypic BRAF^V600E^ status did not always correlate with BRAF^V600E^ protein expression. Mel526 (BRAF^V600E+^ by PCR) did not express altered protein, and this coincided with the lack of sensitivity to BRAFi (Additional file 1: Fig. S1; Table S1).

These cell lines were also assessed for the expression of surface TNFR1 and TNFR2. While TNFR1 was ubiquitously expressed on all the melanoma cell lines evaluated, TNFR2 was expressed on M21, A375, SK-Mel-37 and M255 cells. Both receptors were expressed at various densities (Fig. 1). As solTNF-mediated upregulation of CD271 (low affinity nerve growth factor receptor; p75^NTR^) has been reported to mediate resistance to MAPKi by BRAF^V600E+^ melanomas [10, 11], we also measured the expression of this receptor by flow cytometry. CD271 expression levels directly correlated with TNFR1 and, especially, TNFR2 expression observed on M21, A375, SK-Mel-37 and M255 cell lines (Fig. 1; Additional file 1: Fig. S2).

### solTNF induces MAPKi resistance in BRAF.^V600E+^ melanoma cell lines that co-express TNFR1 and TNFR2

**Fig. 2:**
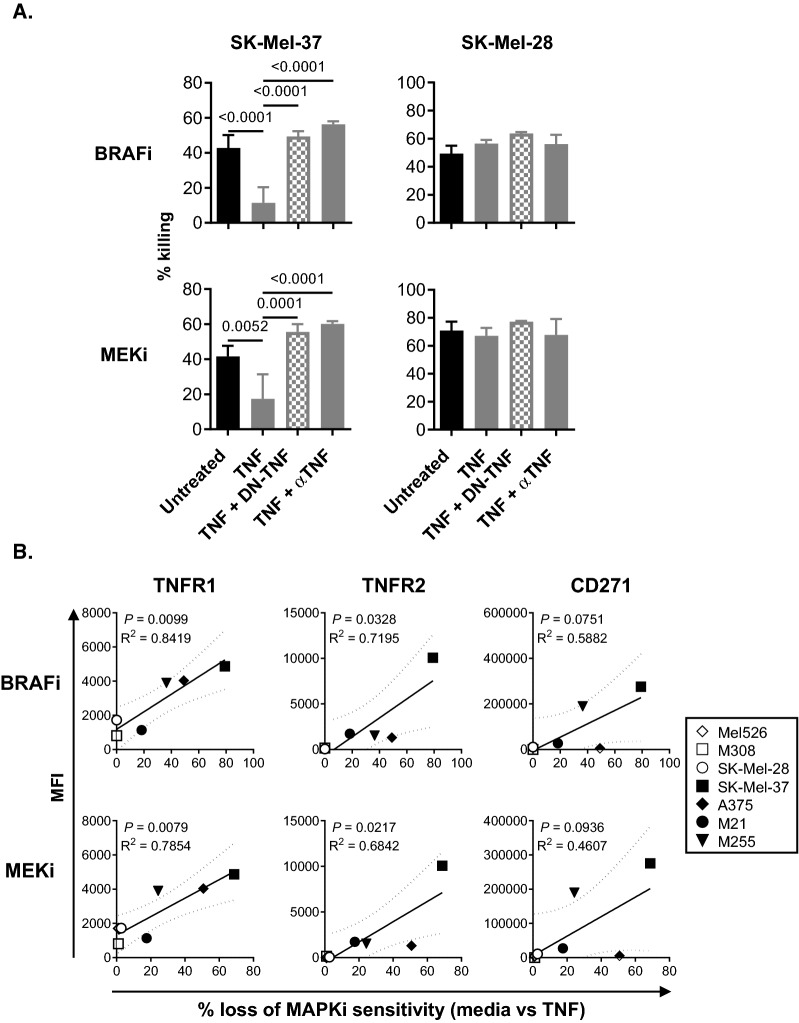
solTNF induces MAPKi resistance in BRAF^V600E+^ melanoma cell lines that co-express TNFR1 and TNFR2. **A** BRAF.^V600E+^ SK-Mel-38 and SK-Mel-28 melanoma cell lines were cultured alone or with rhTNF (TNF) for 48 h. Some of the cells stimulated with rhTNF were co-cultured in the presence of selective solTNF inhibitor (INB03; DN-TNF) or non-selective TNF-blocking antibody (αTNF). **B** Calculated loss of sensitivity to MAPKi in response to rhTNF [% Loss of MAPKi sensitivity (media vs TNF)] was correlated to TNFR1, TNFR2 or CD271 mean fluorescent intensity (MFI) values measured in Fig. 1. Experiments in (**A**) and (**B**) were performed in quadruplicates. Data shown represent mean values and whiskers represent standard error. *p ≤ 0.05

The ability of rhTNF (mimicking solTNF) to promote MAPKi resistance was initially tested using one TNFR1^high^ TNFR2^high^ CD271^high^ (SK-Mel-37) and one TNFR1^low^ TNFR2^−^ CD271^−^ (SK-Mel-28) BRAF^V600E+^ cell line (Fig. 2A). These cells displayed dramatic differences in their ability to acquire resistance to BRAFi and MEKi in response to solTNF treatments. While SK-Mel-37 cells displayed 69–79% decrease in their sensitivity to MAPKi, SK-Mel-28 was unaffected (Fig. 2A). Neutralization of TNF using a monoclonal antibody or selective solTNF inhibitor, DN-TNF [19], led to abrogation of TNF-driven effects (Fig. 2A). When all of the BRAF^V600E+^ cell lines were evaluated, their ability to acquire resistance to BRAFi and MEKi statistically correlated with TNFR1 and TNFR2 expression levels, but not with CD271 (Fig. 2B). These data suggested that only BRAF^V600E+^ melanoma cell lines co-expressing TNFR1 and TNFR2 can acquire resistance to MAPKi in response to solTNF exposure.

### Co-expression of TNFR1 and TNFR2 on melanomas is required for solTNF-induced MAPKi resistance

**Fig. 3:**
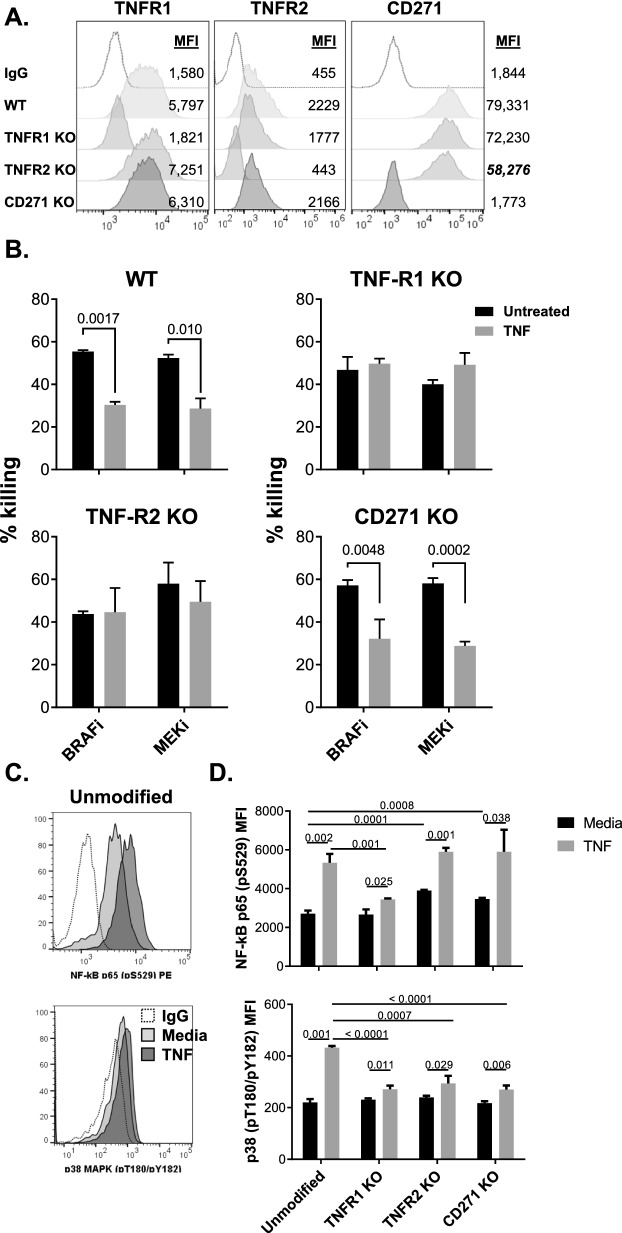
Co-expression of TNFR1 and TNFR2 on melanomas is required for solTNF-induced MAPKi resistance. CRISPR/Cas9-mediated TNFR1, TNFR2 and CD271 knockout (KO) variants of Sk-Mel-37 cell line were generated. **A** The effectiveness of gene alterations was confirmed by flow cytometry. **B** KO cell variants were compared to the unaltered “wild-type” (WT) cell line for their ability acquire resistance to BRAFi and MEKi in response to rhTNF stimulation using the MTT assay. Experiments were performed in quadruplicates. Data shown represent mean values of quadruplicate tests and whiskers represent standard error. *p ≤ 0.05. **C**, **D** Sk-Mel-37 WT, TNFR1, TNFR2 and CD271 KO cell lines were cultured in the presence or absence of solTNF for 15 min. Subsequently, cells were evaluated for phosphorylated NF-kB [phos-NF-kB p65(S536)] and p38 MAPK (pT180/pY182) levels by flow cytometry. Staining examples (**C**) and summary of the data (**D**) are shown. Data are representative of three independent experiments

To explore the roles of TNFR1, TNFR2 and CD271 in solTNF-induced resistance to MAPKi, we individually knocked out (KO) each receptor on SK-Mel-37 cells (Fig. 3A). When comparing TNFR expression patterns on the three cell line variants, we observed that TNFR2 KO cells had 27% lower expression of CD271 (Fig. 3A), suggesting that constitutive TNFR2 signaling may regulate spontaneous expression of CD271 on melanomas. Each of the cell variants, as well as the unmodified SK-Mel-37 (WT), were stimulated for 72 h with solTNF, after which their sensitivities to MAPKi were evaluated. In comparison to WT, both TNFR1 KO and TNFR2 KO lost the ability to acquire resistance to either of the MAPKi, while CD271 KO did not (Fig. 3B), suggesting that TNFR1 and TNFR2 were the critical components of solTNF-mediated resistance to MAPKi in melanoma cells.

### solTNF-induced NF-kB signaling is mediated exclusively via TNFR1, while p38 MAPK signaling is mediated through TNFR1, TNFR2 and CD271

NF-κB phosphorylation has been reported to be the central signaling pathway associated with solTNF-induced MAPKi resistance in BRAF^V600E^ melanomas [10]. Consequently, we evaluated how phosphorylation of NF-κB was affected in WT, TNFR1 KO, TNFR2 KO and CD271 KO cells following solTNF treatments. NF-κB phosphorylation was induced in all but TNFR1 KO cells (Fig. 3C, D), supporting the notion that solTNF-induced signaling is mediated exclusively via TNFR1. It was also of interest to evaluate whether MAPK signaling was also bypassed by the solTNF stimuli. While solTNF treatment did lead to increased phosphorylation of p38 MAPK in WT cells, this signaling pathway was absent in all the SK-Mel-37 KO variants, including CD271 (Fig. 3C, D). As CD271 KO cells do acquire resistance to MAPKi in response to solTNF treatments, it can be concluded that the p38 MAPK signaling pathway is not critical to this acquired resistance mechanism.

### Forced expression of TNFR2 enables solTNF-induced MAPKi resistance in a previously unresponsive cell line

**Fig. 4:**
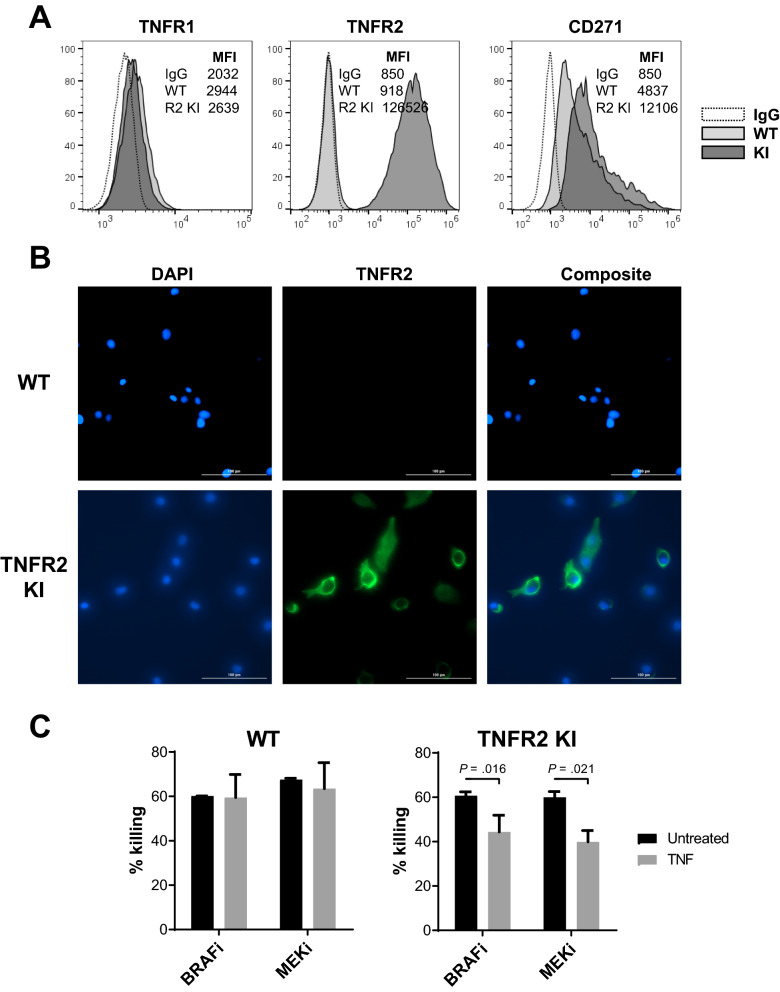
Forced TNFR2 expression sensitizes BRAFV^600E+^ melanoma to solTNF-induced resistance to MAPKi. TNFR2 knockin (KI) SK-Mel-28 variant was generated. TNFR profile was evaluated by **A** flow cytometry and **B** immunofluorescence microscopy. **C** Unmodified “wild-type” (WT) and stably-transfected TNFR2.^+^ SK-Mel-28 cells (TNFR2 KI) were cultured in the presence or absence of solTNF and tested for their sensitivity to BRAFi and MEKi-mediated killing. Data shown represent mean values of triplicate tests and whiskers represent standard error. *p ≤ 0.05

To validate our observations generated with loss-of-function experiments, we generated a stable TNFR2-expressing SK-Mel-28 cell line that displayed TNFR2 translocation to the plasma membrane (TNFR2 KI; Fig. 4A, B). Forced expression of TNFR2 led to 25-fold increase in CD271 expression on the cells, supporting the idea that constitutive TNFR2 signaling may partially drive spontaneous CD271 expression. When compared to the TNFR2^−^ maternal cell line, TNFR2^+^ variant displayed 27–33% decrease in its sensitivity to MAPKi following solTNF treatments (Fig. 4C). These results support the idea that TNFR2 co-expression with TNFR1 is necessary for solTNF-mediated resistance to MAPKi.

### Neutralization of solTNF abrogates macrophage-mediated resistance to MAPKi

**Fig. 5:**
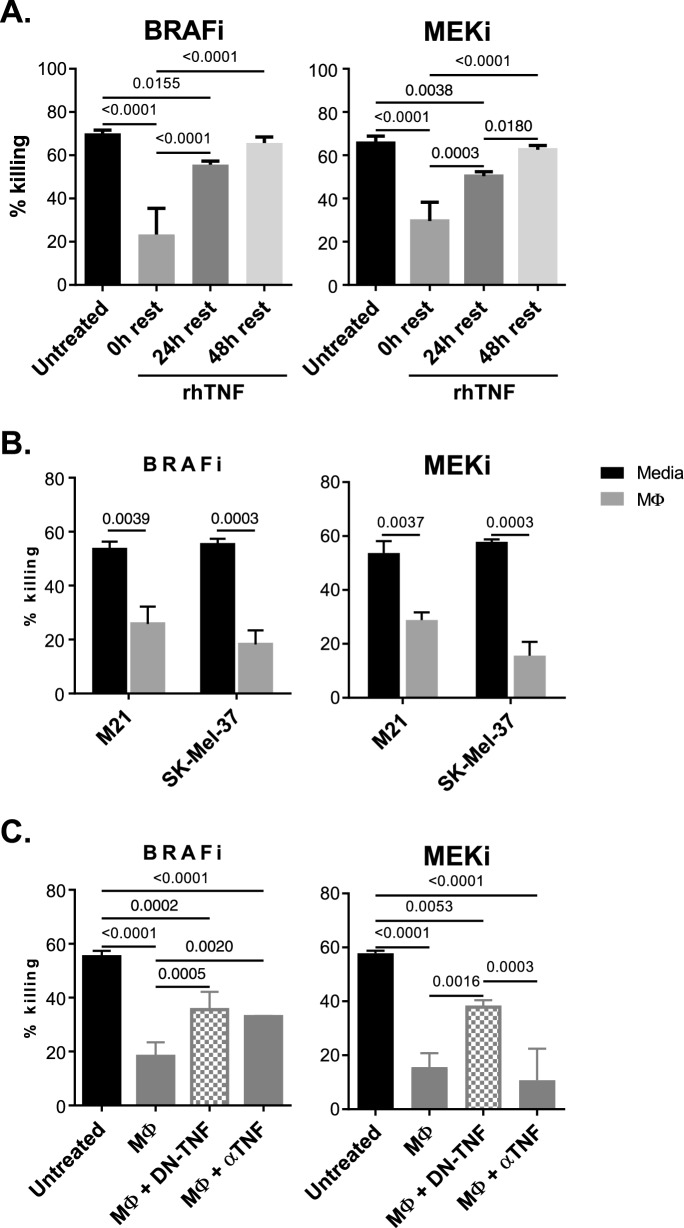
solTNF-driven resistance to MAPKi is transient and can be neutralized using solTNF-targeting biologics. **A** SK-Mel-37 cells were treated with solTNF for 72 h, after which they were rested in solTNF-free medium for 0 h, 24 h or 48 h and tested for their sensitivity to MAPKi. **B** Activated macrophages (MΦ) were seeded in 0.4 μm transwell inserts and placed into wells containing M21 or SK-Mel-37 cells. After 48 h co-culture, melanoma cell lines were collected and tested for MAPKi sensitivity. **C** The ability of DN-TNF and αTNF to abrogate macrophage TNF-induced resistance to MAPKi was tested. Data are representative of three independent experiments. Experiments (**A–C**) were performed in quadruplicates. Data shown represent mean values and whiskers represent standard error. *p ≤ 0.05

To explore potential clinical relevance of these data, we first wanted to establish whether exposure to solTNF leads to permanent resistance to MAPKi by BRAF^V600E+^ melanoma or whether these effects are reversible. To test this, SK-Mel-37 WT cells were treated for 72 h with solTNF, after which solTNF was washed out. Melanoma sensitivity to MAPKi was measured 0, 24 and 48 h after solTNF removal (Fig. 5A). We observed that solTNF-driven effects were reversible as 48 h incubation of cells in solTNF-free media completely restored cell sensitivity to MAPKi. As macrophages are one of the key drivers of BRAF^V600E+^ melanoma resistance to MEKi [10, 23], it was also critical to explore whether TNFR2^+^ cell lines could favorably respond to activated monocyte-derived macrophages (Additional file 1: Fig. S3A). Using transwell co-cultures, M21 and SK-Mel-37 cell lines were tested for their ability to acquire resistance to BRAFi and MEKi in response to factors released by activated macrophages. Both cell lines acquired resistance to BRAFi and MEKi in response to pre-treatment with macrophages. M21 displayed ~ 49%, while SK-Mel-37 showed 65–75% decrease in sensitivity to MAPKi (Fig. 5B). Next, using SK-Mel-37 cells, we explored whether treatments with anti-TNF antibody, which neutralizes both tmTNF and solTNF, or DN-TNF, which specifically neutralizes solTNF, could abrogate macrophage-mediated effects. For BRAFi resistance, we observed that both anti-TNF and DN-TNF treatments led to partial, but significant abrogation of macrophage-induced effects (67% decreased sensitivity post macrophage treatment vs. 41–42% decreased sensitivity in the presence of macrophages and TNF blockers; Fig. 5C). Interestingly, for MEKi, while anti-TNF antibody was ineffective, DN-TNF treatment mitigated macrophage-mediated resistance (70% decreased sensitivity post macrophage treatment vs. 32% decreased sensitivity in the presence of macrophages and DN-TNF; Fig. 5C). Partial effectiveness of TNF inhibitors to neutralize macrophage-induced resistance to MAPKi by BRAF^V600E+^ melanoma could be attributed to the ability of macrophages to release solTNF and VEGF (Additional file 1: Fig. S3B), two factors central to their MEKi-promoting functions [10, 23].

### TNFR2 is commonly expressed by BRAF^V600E+^ metastatic melanoma

**Fig. 6:**
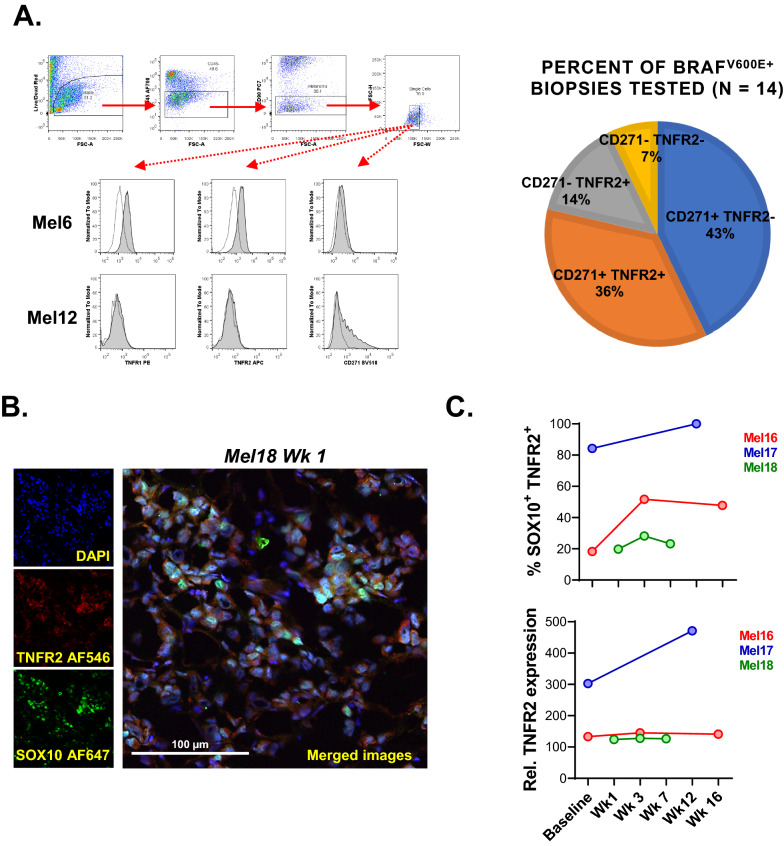
BRAFV^600E+^ melanomas commonly express TNFR2. **A** Single-cell melanoma suspensions derived from BRAF^V600E+^ metastatic lesions were evaluated for TNFR1, TNFR2 and CD271 expression by multi-color flow cytometry. Cells were stained for Live/Dead Red (dead cell exclusion), CD45 (leukocyte exclusion), CD90 (fibroblast exclusion), TNFR1, TNFR2 and CD271. A gating strategy and staining examples from two melanoma patients are shown on the left. Summary of TNFR2 and CD271 distribution observed in 16 patients harboring BRAF^V600E+^ melanoma (shown as percentages) is depicted on the right. **B**, **C** Frequencies of SOX10^+^ TNFR2^+^ melanoma, as well as relative TNFR2 expression levels on SOX10^+^ melanoma cells were elevated by immunofluorescence microscopy using biopsies acquired from patients receiving vemurafenib and high dose IFN-α2b therapy. Data were extrapolated from high resolution whole section scans. Vemurafenib was administered orally for three weeks, after which time the patients received high-dose IFN-α2b concurrently with vemurafenib

To translate our observations into the clinical setting, single-cell melanoma suspensions derived from BRAF^V600E+^ metastatic lesions (Additional file 1: Table S2) were evaluated for TNFR1, TNFR2 and CD271 expression by multi-color flow cytometry (Fig. 6A). 50% of BRAF^V600E+^ metastatic melanomas tested expressed TNFR2, most of which co-expressed CD271. 43% of all tested primary melanoma cells expressed CD271, but not TNFR2. For functional tests, we generated two primary BRAF^V600E+^ melanoma cell lines: a TNFR1^low^ TNFR2^−^ CD271^high^ and TNFR1^low^ TNFR2^low^ CD271^low^ cell line (Additional file 1: Fig. S4A). Both cell lines were responsive to BRAFi and MEKi, however only the cell line expressing low levels of TNFR2 showed moderate acquisition of resistance to BRAFi in response to solTNF pre-treatment (Additional file 1: Fig. S4B).

### Frequency of TNFR2-expression melanoma cells increases following vemurafenib treatments

To explore whether TNFR2 expression can be modulated in response to MAPKi-based therapies, we performed immunofluorescence microscopy analysis of biopsies excised from patients receiving vemurafenib and high dose IFN-α2b therapy (Fig. 6B, C). We were able to identify three patients with sufficient viable tissues collected from at least two timepoints. Vemurafenib was given orally for three weeks. At week 3, high-dose IFN-α2b was administered concurrently with vemurafenib. Frequencies of SOX10^+^ TNFR2^+^ melanoma were elevated in all three patients by week 3, prior to the administration of IFN-α2b therapy. In one patient (Mel17) we also observed increased relative TNFR2 expression levels. These data suggest that vemurafenib therapy may select for TNFR2^+^ melanoma or induce TNFR2 expression on melanoma.

### TNFR2 expression is not directly induced on BRAF^V600E+^ melanomas by MAPKi

To evaluate whether TNFR2 upregulation on human BRAF^V600E+^ melanomas is directly mediated by MAPKi, we treated the TNFR2-negative SK-Mel-28 cell line with BRAFi or MEKi. IFN-γ, a known inducer of TNFR2 expression on human melanomas [13], was tested in parallel. BRAFi and MEKi treatments did not induce TNFR2 expression, while IFN-γ did (Additional file 1: Fig. S5). These results support the idea that TNFR2 expression is not directly induced by MAPKi.

## Discussion

TNF is a growth factor for melanoma that blocks MAPKi-induced apoptosis in BRAF^V600E+^ melanomas via NF-kB signaling [9]. Smith, M.P. et al. have reported that MAPKi therapy leads to increased infiltration of melanoma by TAM that, through TNF release, induce MAPKi resistance and promote cell survival via enhanced NF-κB signaling and upregulation of downstream lineage transcription factor MITF [10]. In a recent mouse study, MAPKi were confirmed to promote melanoma inflammation via pyroptosis, exemplified by the influx of TNF-producing T cells [24]. Cumulatively, these publications indicate that MAPKi promote a TNF-rich TME where selective inhibition of TNF-mediated signaling may enhance their therapeutic benefits. While studies have identified TNF as a potent inducer of MAPKi resistance in BRAF^V600E+^ melanomas, the prevalence of this adaptive resistance mechanism remains unclear.

Functional solTNF and tmTNF are homotrimeric molecules. solTNF is formed when the tmTNF extracellular domain is cleaved by the TNF-alpha-converting enzyme (TACE/ADAM17) [25]. solTNF and tmTNF preferentially activate TNFR1 and TNFR2, respectively [12]. tmTNF binds more strongly to TNFR2 than does solTNF due to the formation of a tight trimer that is unable to dissociate from the receptor [26]. solTNF can bind both TNF receptors, with TNFR1 having a significantly higher affinity for it (K_d_ = 1.9 × 10^−11^ M) than TNFR2 (K_d_ = 4.2 × 10^−10^ M) [27]. While TNFR1 mediates apoptosis and cell survival/cytokine secretion, TNFR2 promotes cell survival/cytokine secretion [12]. Previously it was reported that TNFR2 can be expressed on human melanomas and that it is IFN-γ-inducible [13]. As selective MAPKi were reported to promote T and NK cell infiltration into human metastatic melanoma [28, 29], it is feasible that IFN-γ- and TNF-producing T and NK cells can modulate TNFR2 expression by and induce MAPKi resistance in BRAF^V600E+^ metastatic melanomas.

The prevalence and function of TNFR2 on melanomas, especially in the context of BRAF-mutated melanomas, is poorly understood. Our data indicate that TNFR2 expression on melanomas can be controlled by cell intrinsic and extrinsic factors. We show that TNFR2 is constitutively expressed on 4/7 melanoma cell lines and 50% of BRAF^V600E+^ metastatic lesions tested. Critically, we show that TNFR2 expression is elevated on melanoma cells in patients treated with MAPKi, which suggests an acquired resistance mechanism that is mediated either through enrichment of melanoma cells that constitutively express TNFR2 or through induction of TNFR2 expression. Our in vitro studies suggest that MAPKi do not directly induce TNFR2 expression on melanomas, but that IFN-γ does. Since MAPKi promote increased tumor infiltration and IFN-γ secretion by both CD4^+^ and CD8^+^ T cells in BRAF^V600E+^ melanoma animal models [30, 31], it is feasible that TNFR2 upregulation on patient melanomas results from a negative feedback loop mediated by MAPKi-enhanced Th1-type tumor-specific T cell responses. Consequently, future studies of this resistance mechanism should include more holistic approaches that would explore the roles of CD4^+^ and CD8^+^ T cells in this resistance mechanism.

Our data indicate that solTNF induces NF-κB signaling in melanoma via selective triggering of TNFR1 and not TNFR2, confirming the pre-established solTNF-TNFR1 signaling axis [32]. However, while TNFR2 does not appear to mediate NF-κB signaling in response to solTNF, it does mediate p38 MAPK phosphorylation and plays a vitally supportive role for solTNF-mediated TNFR1 signaling and induction of MAPKi resistance in melanoma. This observation can potentially be attributed to ligand passing, a process by which TNFR2 enhances TNFR1 signaling by regulating the rate of solTNF association with TNFRl through rapid ligand association and dissociation [33]. While ligand passing has been described as a necessary mechanism for maximum responsiveness to solTNF by various leukocyte subsets, including neutrophils [34], it has not been reported in melanomas.

Previous reports strongly implicate solTNF as the key form of TNF that mediates MAPKi resistance [9–11]. They suggest that solTNF-mediated NF-kB signaling drives MAPKi resistance via CD271 [11]. Our study indicates that the role of CD271 in MAPKi resistance is more ambiguous than previously thought [11]. We show that co-expression of TNFR1 and TNFR2, but not CD271, is required for BRAF^V600E+^ melanomas to acquire resistance to MAPKi in response to solTNF exposure. One possible reason why CD271 has previously been implicated in MAPKi resistance is due to its common co-expression with TNFR1 and TNFR2, and because TNFR2 partially drives CD271 upregulation on cultured BRAF^V600E+^ melanoma cell lines. Interestingly, concurrent expression of CD271 and TNFR2 is not as common among BRAF-mutant melanomas in vivo*,* which suggests that CD271 expression can also be modulated by the TME. This could be attributed to the fact that melanoma exposed to proinflammatory stimuli can upregulate CD271 [35].

Since solTNF has been implicated as one of the key exogenous factors that promotes resistance to MAPKi by BRAF-mutant melanoma [9, 10], addition of NF-κB signaling pathway inhibitors (e.g. BMS-345541) or TNF antagonists (e.g. etanercept, adalimumab, infliximab) has been proposed to enhance MAPKi-based therapies to improve their clinical efficacy. NF-κB inhibitors are a poor choice for the clinic as this signaling pathway is central to a variety of vital functions, including cell survival, cytokine secretion and effective antitumor immune response and is not specific to solTNF [36]. While clinically safe [37], the current generation of TNF blockers are also a suboptimal choice as they non-specifically block both solTNF and tmTNF [36]. Dendritic cell (DC)/natural killer (NK)-cell crosstalk, which drives Th1-type anti-tumor immunity and defines ICB-responsive TME [38], is exclusively mediated via tmTNF-TNFR2 [21, 39, 40]. In contrast, inflammation-driven autoimmune diseases are mediated by solTNF [40]. Sequestration of TNF has been tested in Phase I and II clinical cancer trials with TNF antagonists as single agents. Infliximab (chimeric monoclonal antibody) and etanercept (TNFR2-Fc construct) were tested against multiple types of cancer, including melanoma with limited success [41–46]. Conversely, multiple studies have shown that patients with autoimmune disorders who are treated with the current generation of general TNF inhibitors (e.g., etanercept, infliximab, adalimumab) are at increased risk of tuberculosis and certain malignancies including melanoma [47–49]. These reports may indicate that tmTNF, which is sequestered along with solTNF, plays an important role in cellular immunity that controls the development of melanoma in high-risk individuals. They also stress the need to develop novel agents that specifically target pro-carcinogenic solTNF.

One such agent is DN-TNF, a selective inhibitor of solTNF originally developed as a next generation TNF blocker [50]. DN-TNF is a PEGylated protein that selectively forms non-functional heterotrimers with nascent solTNF [21, 50]. Its potency was confirmed in vivo in multiple autoimmune models, including arthritis where it was shown to attenuate the disease without suppressing cellular immunity to infection [51]. We have shown that DN-TNF can block chemically induced carcinogenesis, accumulation of MDSCs and immunosuppression in mice [46]. In the present study, we tested the ability of DN-TNF to block MAPKi resistance mediated by recombinant and macrophage-derived solTNF. DN-TNF treatment completely blocked recombinant solTNF-induced resistance to MAPKi, however it only led to partial, albeit significant abrogation of macrophage-induced effects. This could be contributed to the fact that activated macrophages also release VEGF that can drive MAPKi resistance [52]. Finally, we have previously reported that DN-TNF treatments decrease splenic macrophage frequencies in tumor-bearing mice [46]. Consequently, addition of DN-TNF to MAPKi-based therapies may benefit patients harboring BRAF^V600E+^ melanoma in two ways: by directly blocking solTNF-driven drug resistance and by decreasing macrophage frequencies in the TME.

## Conclusions

We report that only a subset of melanomas acquire MAPKi resistance in response to TNF exposure. More importantly, we identify TNFR2 as a key component to this mechanism and provide evidence that it can potentially be used as a biomarker of melanomas that might benefit from combination MAPKi and TNF-targeting therapies.

## Supplementary Information

**Additional file 1: Table S1.** Melanoma cell line phenotypes and MAPKi sensitivities. **Table S2.** Melanoma patient characteristics. **Figure S1.** BRAFV600E+ melanoma cell lines express various levels of mutant BRAF protein. PCR-generated BRAFV600E mutation status of human melanoma cell lines utilized in this study was confirmed by flow cytometry (BRAFV600E Antibody VE1; Roche). **Figure S2.** Correlation of TNFR1, TNFR2 and CD271 expression levels on BRAFV600E+ melanoma cell lines. Expression levels of the three receptors adjusted for respective IgG controls (Fig. 1) were evaluated by linear correlation. **Figure S3.** Generation and activation of monocyte-derived macrophages. Human monocyte-derived macrophage (MΦ) phenotype was confirmed by A) flow cytometry (CD14+CD68+CD80+CD86+) and B) their ability to release solTNF and VEGF in response to LPS + IFNγ activation. **Figure S4.** TNFR2+ primary BRAFV600E+ melanoma cell line can acquire resistance to BRAFi in response to solTNF treatment. (A) Primary melanoma cell lines were generated from two BRAFV600E+ melanoma patient biopsies. Their TNFR1, TNFR2 and CD271 profiles were evaluated by flow cytometry. (B) Early passage cell lines (10 passages or less) cultured in the presence or absence of solTNF were tested for their sensitivity to BRAFi and MEKi-mediated cytotoxicity. Data shown represent mean values of quadruplicate tests and whiskers represent standard error. *p ≤ 0.05. **Figure S5.** TNFR2 expression on BRAFV600E+ melanoma is upregulated in response to IFN-γ, but not MAPKi. SK-Mel-28 cell line was cultured for 48 h in the presence or absence of IFN-γ (1000 IU/ml), 0.5 µM BRAFi (0.5 µM) and MEKi (0.2 µM). Subsequently, cells were collected and stained for TNFR2 by 2-step staining as previously described. Data shown are representative of three independent experiments.

## Data Availability

The datasets used and/or analyzed during the current study are available from the corresponding author on reasonable request.

## Abbreviations

BRAF: V-raf murine sarcoma viral oncogene homolog B1
BRAFi: BRAF inhibitor
DN-TNF: Dominant-negative tumor necrosis factor
E.D._50_: Median effective dose
IFN-α2b: Interferon-α2b
IFN-γ: Interferon-γ
LPS: Lipopolysaccharide
M-CSF: Macrophage colony-stimulating factor
MAPK: Mitogen-activated protein kinase
MAPKi: MAPK pathway inhibitor
MEKi: MAPK kinase inhibitor
MTT: 3-(4,5-Dimethylthiazol-2-yl)-2,5-diphenyltetrazolium bromide
PBMC: Peripheral blood mononuclear cells
PE: Phycoerythrin
rhTNF: Recombinant human TNF
solTNF: Soluble tumor necrosis factor
TAM: Tumor-associated macrophage
TME: Tumor microenvironment
tmTNF: Transmembrane tumor necrosis factor
TNF: Tumor necrosis factor
TNFR1: Type-1 TNF receptor
TNFR2: Type-2 TNF receptor
