# Supplementary material for: Co-expression of TNF receptors 1 and 2 on melanomas facilitates soluble TNF-induced resistance to MAPK pathway inhibitors
Source: J Transl Med. 2022 Jul 25;20:331. doi: 10.1186/s12967-022-03538-w (PMC9310383; doi:10.1186/s12967-022-03538-w)
Supplement: Supplementary file 1 — Additional file 1: Table S1. Melanoma cell line phenotypes and MAPKi sensitivities. Table S2. Melanoma patient characteristics. Figure S1. BRAFV600E+ melanoma cell lines express various levels of mutant BRAF protein. PCR-generated BRAFV600E mutation status of human melanoma cell lines utilized in this study was confirmed by flow cytometry (BRAFV600E Antibody VE1; Roche). Figure S2. Correlation of TNFR1, TNFR2 and CD271 expression levels on BRAFV600E+ melanoma cell lines. Expression levels of the three receptors adjusted for respective IgG controls (Fig. 1) were evaluated by linear correlation. Figure S3. Generation and activation of monocyte-derived macrophages. Human monocyte-derived macrophage (MΦ) phenotype was confirmed by A) flow cytometry (CD14+CD68+CD80+CD86+) and B) their ability to release solTNF and VEGF in response to LPS + IFNγ activation. Figure S4. TNFR2+ primary BRAFV600E+ melanoma cell line can acquire resistance to BRAFi in response to solTNF treatment. (A) Primary melanoma cell lines were generated from two BRAFV600E+ melanoma patient biopsies. Their TNFR1, TNFR2 and CD271 profiles were evaluated by flow cytometry. (B) Early passage cell lines (10 passages or less) cultured in the presence or absence of solTNF were tested for their sensitivity to BRAFi and MEKi-mediated cytotoxicity. Data shown represent mean values of quadruplicate tests and whiskers represent standard error. *p ≤ 0.05. Figure S5. TNFR2 expression on BRAFV600E+ melanoma is upregulated in response to IFN-γ, but not MAPKi. SK-Mel-28 cell line was cultured for 48 h in the presence or absence of IFN-γ (1000 IU/ml), 0.5 µM BRAFi (0.5 µM) and MEKi (0.2 µM). Subsequently, cells were collected and stained for TNFR2 by 2-step staining as previously described. Data shown are representative of three independent experiments. [file 12967_2022_3538_MOESM1_ESM.pptx]

## Slide 1
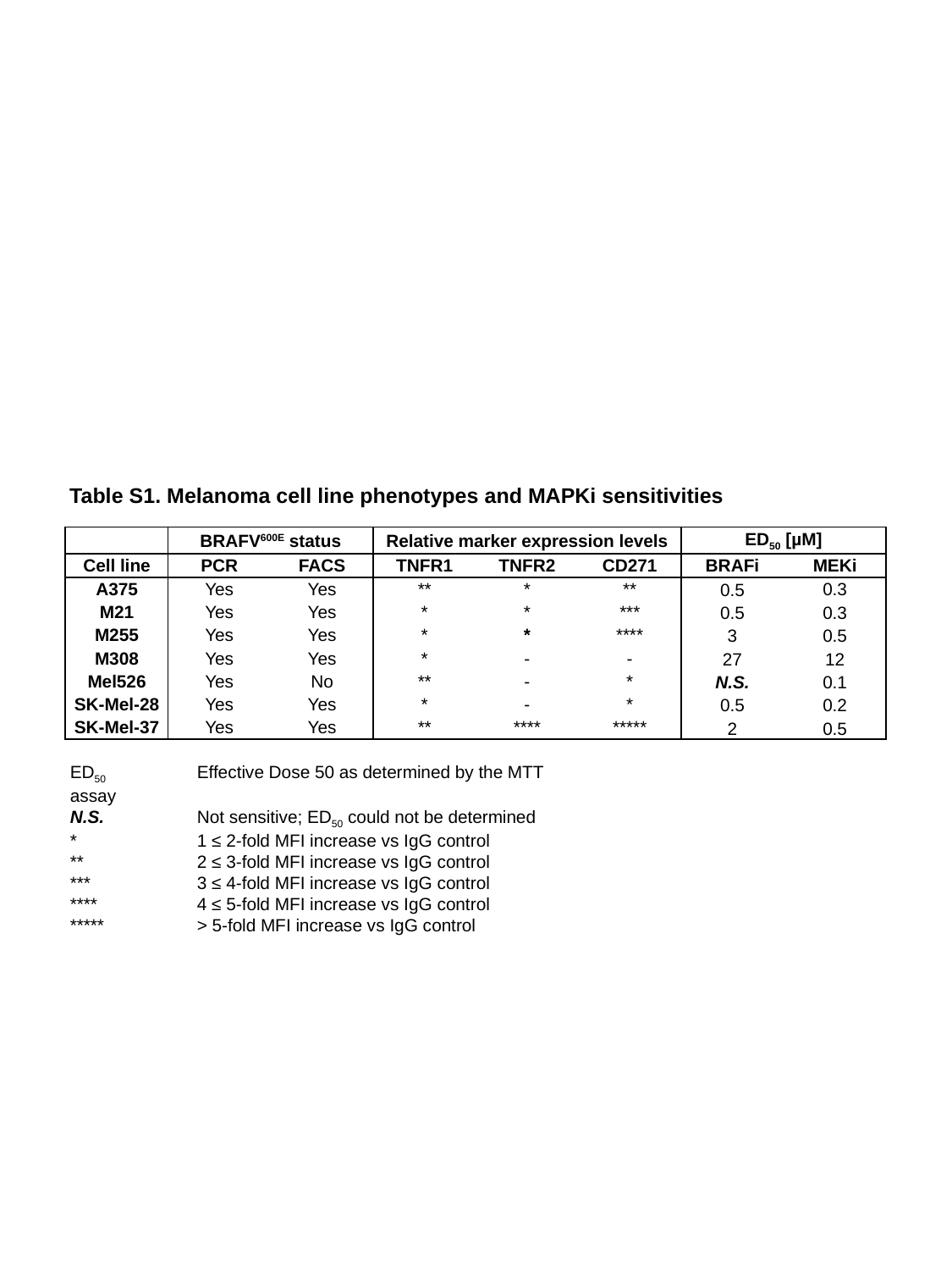

Table S1. Melanoma cell line phenotypes and MAPKi sensitivities
| | BRAFV600E status | | Relative marker expression levels | | | ED50 [µM] | |
| --- | --- | --- | --- | --- | --- | --- | --- |
| Cell line | PCR | FACS | TNFR1 | TNFR2 | CD271 | BRAFi | MEKi |
| A375 | Yes | Yes | \*\* | \* | \*\* | 0.5 | 0.3 |
| M21 | Yes | Yes | \* | \* | \*\*\* | 0.5 | 0.3 |
| M255 | Yes | Yes | \* | \* | \*\*\*\* | 3 | 0.5 |
| M308 | Yes | Yes | \* | - | - | 27 | 12 |
| Mel526 | Yes | No | \*\* | - | \* | N.S. | 0.1 |
| SK-Mel-28 | Yes | Yes | \* | - | \* | 0.5 | 0.2 |
| SK-Mel-37 | Yes | Yes | \*\* | \*\*\*\* | \*\*\*\*\* | 2 | 0.5 |
ED50 	Effective Dose 50 as determined by the MTT assay
N.S. 	Not sensitive; ED50 could not be determined
*	1 ≤ 2-fold MFI increase vs IgG control
**	2 ≤ 3-fold MFI increase vs IgG control
***	3 ≤ 4-fold MFI increase vs IgG control
****	4 ≤ 5-fold MFI increase vs IgG control
***** 	> 5-fold MFI increase vs IgG control

## Slide 2
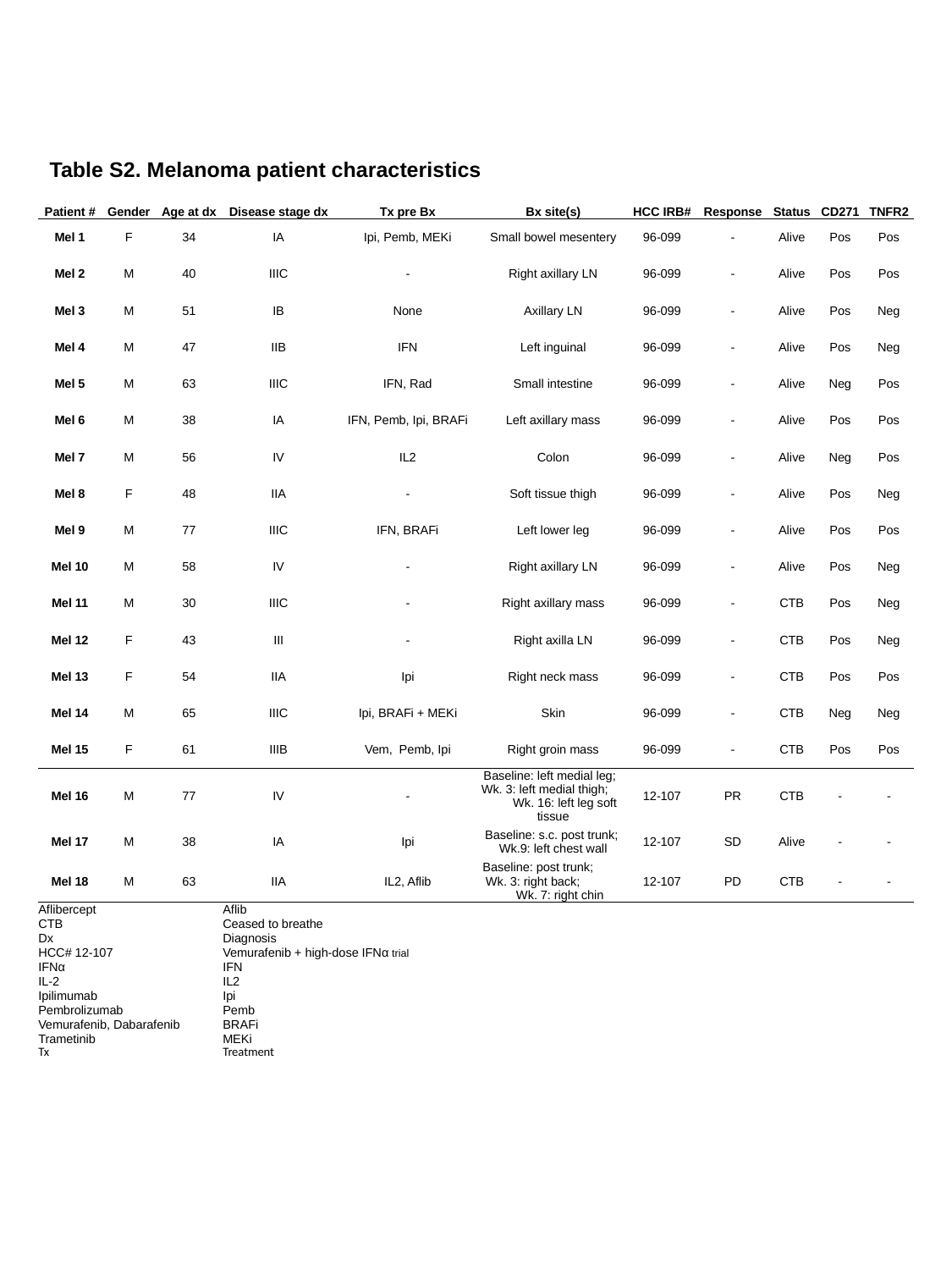

Table S2. Melanoma patient characteristics
| Patient # | Gender | Age at dx | Disease stage dx | Tx pre Bx | Bx site(s) | HCC IRB# | Response | Status | CD271 | TNFR2 |
| --- | --- | --- | --- | --- | --- | --- | --- | --- | --- | --- |
| Mel 1 | F | 34 | IA | Ipi, Pemb, MEKi | Small bowel mesentery | 96-099 | - | Alive | Pos | Pos |
| Mel 2 | M | 40 | IIIC | - | Right axillary LN | 96-099 | - | Alive | Pos | Pos |
| Mel 3 | M | 51 | IB | None | Axillary LN | 96-099 | - | Alive | Pos | Neg |
| Mel 4 | M | 47 | IIB | IFN | Left inguinal | 96-099 | - | Alive | Pos | Neg |
| Mel 5 | M | 63 | IIIC | IFN, Rad | Small intestine | 96-099 | - | Alive | Neg | Pos |
| Mel 6 | M | 38 | IA | IFN, Pemb, Ipi, BRAFi | Left axillary mass | 96-099 | - | Alive | Pos | Pos |
| Mel 7 | M | 56 | IV | IL2 | Colon | 96-099 | - | Alive | Neg | Pos |
| Mel 8 | F | 48 | IIA | - | Soft tissue thigh | 96-099 | - | Alive | Pos | Neg |
| Mel 9 | M | 77 | IIIC | IFN, BRAFi | Left lower leg | 96-099 | - | Alive | Pos | Pos |
| Mel 10 | M | 58 | IV | - | Right axillary LN | 96-099 | - | Alive | Pos | Neg |
| Mel 11 | M | 30 | IIIC | - | Right axillary mass | 96-099 | - | CTB | Pos | Neg |
| Mel 12 | F | 43 | III | - | Right axilla LN | 96-099 | - | CTB | Pos | Neg |
| Mel 13 | F | 54 | IIA | Ipi | Right neck mass | 96-099 | - | CTB | Pos | Pos |
| Mel 14 | M | 65 | IIIC | Ipi, BRAFi + MEKi | Skin | 96-099 | - | CTB | Neg | Neg |
| Mel 15 | F | 61 | IIIB | Vem, Pemb, Ipi | Right groin mass | 96-099 | - | CTB | Pos | Pos |
| Mel 16 | M | 77 | IV | - | Baseline: left medial leg; Wk. 3: left medial thigh; Wk. 16: left leg soft tissue | 12-107 | PR | CTB | - | - |
| Mel 17 | M | 38 | IA | Ipi | Baseline: s.c. post trunk; Wk.9: left chest wall | 12-107 | SD | Alive | - | - |
| Mel 18 | M | 63 | IIA | IL2, Aflib | Baseline: post trunk; Wk. 3: right back; Wk. 7: right chin | 12-107 | PD | CTB | - | - |
| Aflibercept | | | Aflib | | | | | | | |
| CTB | | | Ceased to breathe | | | | | | | |
| Dx | | | Diagnosis | | | | | | | |
| HCC# 12-107 | | | Vemurafenib + high-dose IFNα trial | | | | | | | |
| IFNα | | | IFN | | | | | | | |
| IL-2 | | | IL2 | | | | | | | |
| Ipilimumab | | | Ipi | | | | | | | |
| Pembrolizumab | | | Pemb | | | | | | | |
| Vemurafenib, Dabarafenib | | | BRAFi | | | | | | | |
| Trametinib | | | MEKi | | | | | | | |
| Tx | | | Treatment | | | | | | | |

## Slide 3
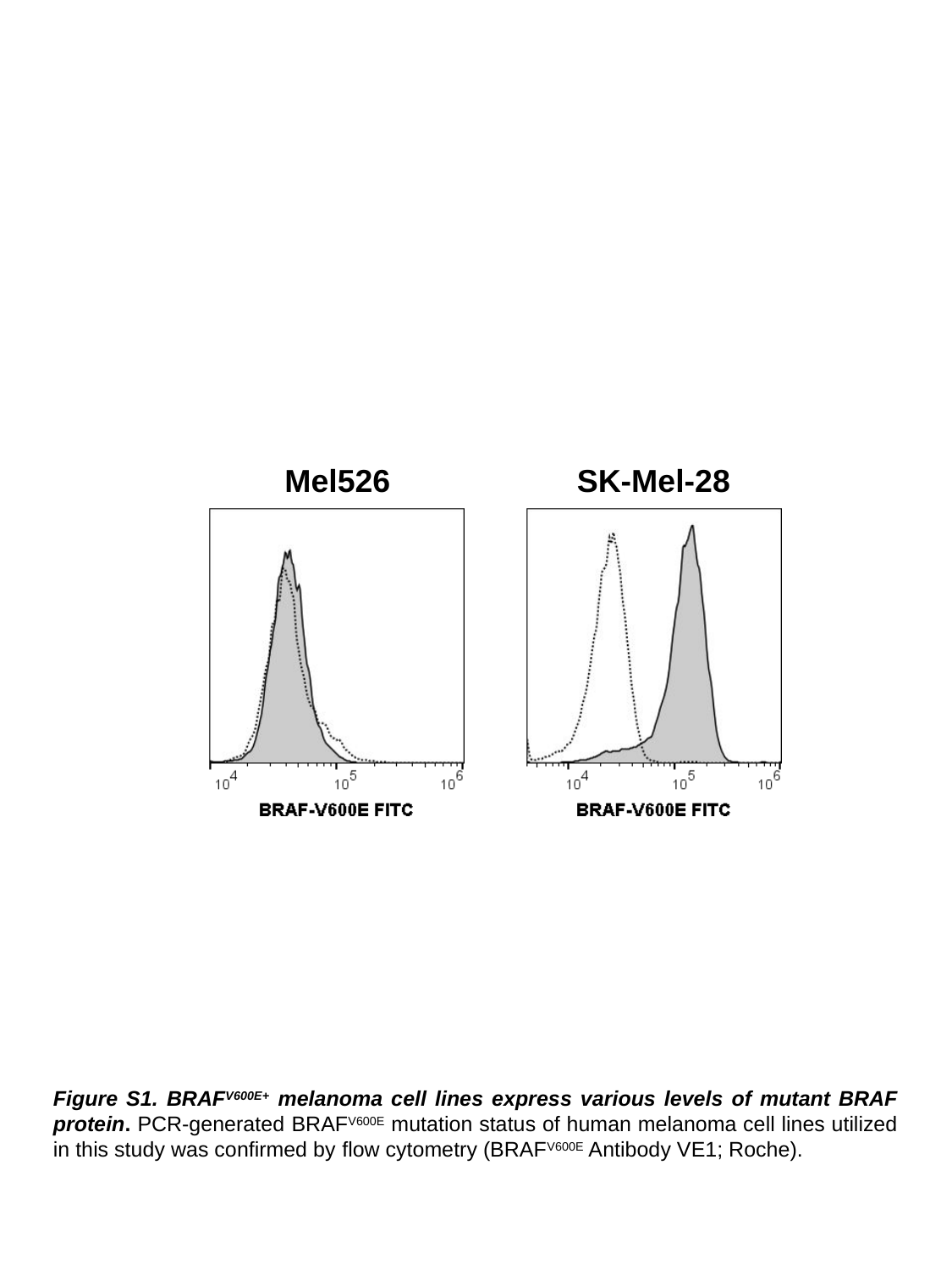

Mel526
SK-Mel-28
Figure S1. BRAFV600E+ melanoma cell lines express various levels of mutant BRAF protein. PCR-generated BRAFV600E mutation status of human melanoma cell lines utilized in this study was confirmed by flow cytometry (BRAFV600E Antibody VE1; Roche).

## Slide 4
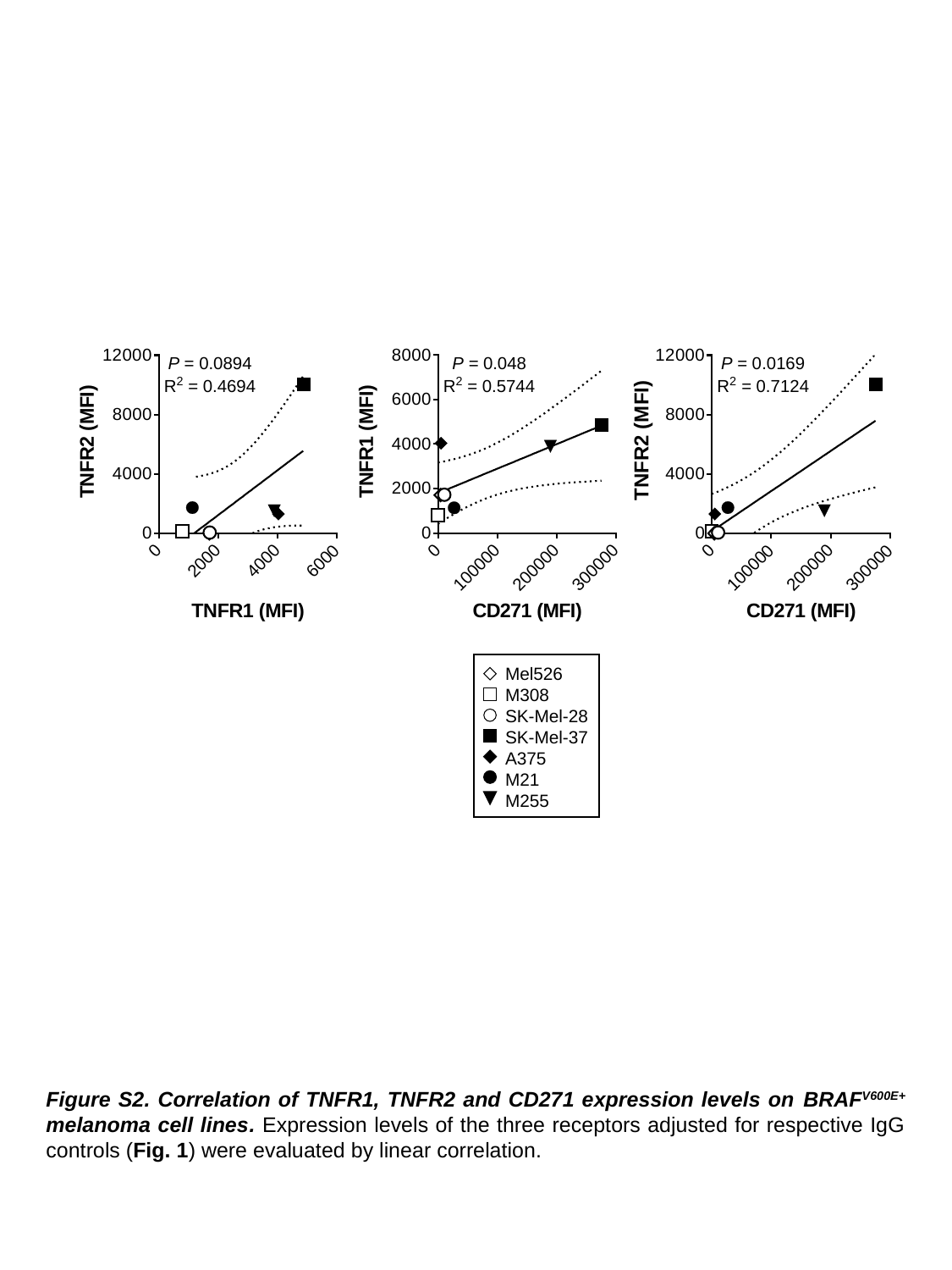

TNFR2 (MFI)
Mel526
M308
SK-Mel-28
SK-Mel-37
A375
M21
M255
Figure S2. Correlation of TNFR1, TNFR2 and CD271 expression levels on BRAFV600E+ melanoma cell lines. Expression levels of the three receptors adjusted for respective IgG controls (Fig. 1) were evaluated by linear correlation.

## Slide 5
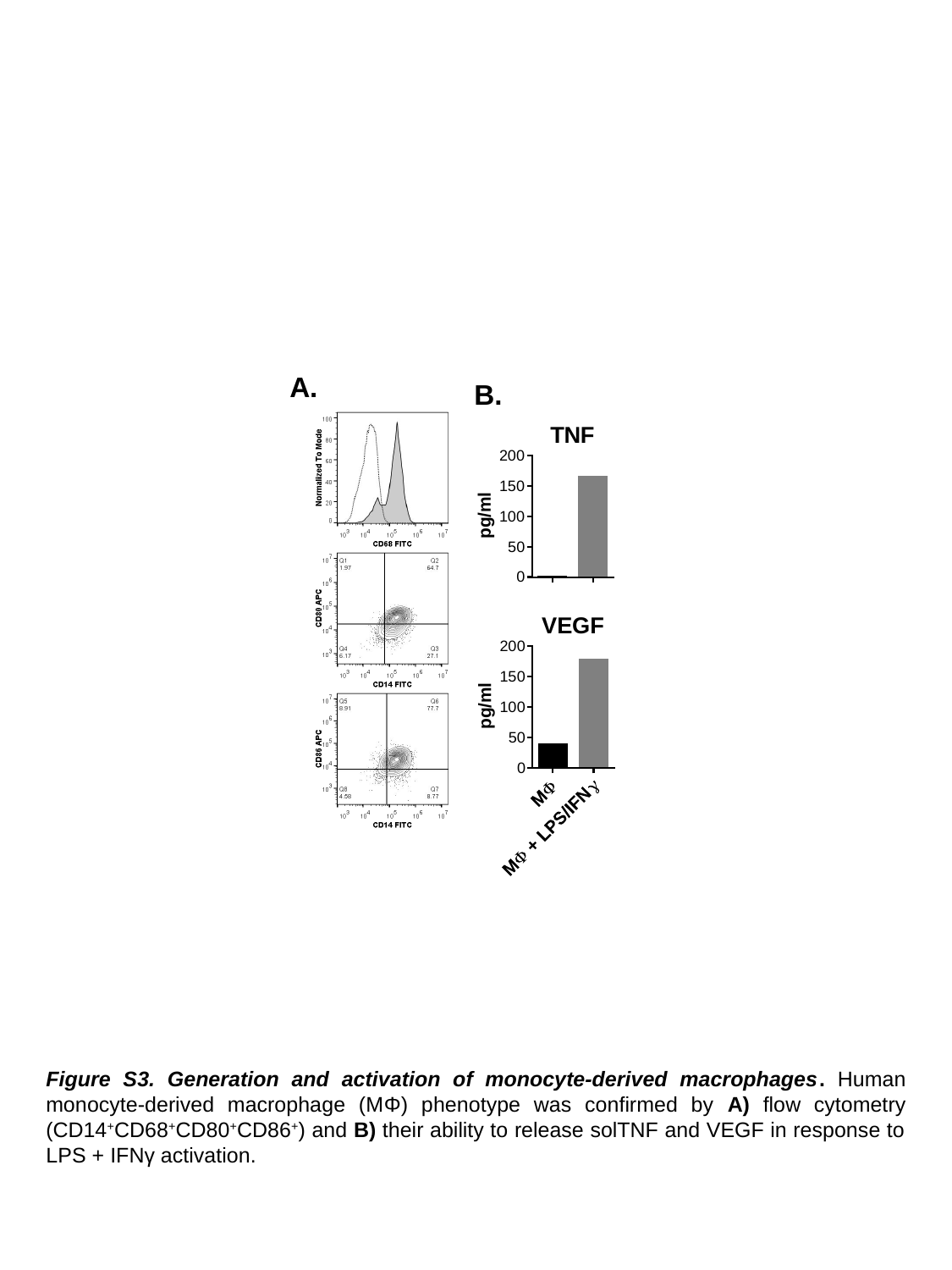

A.
B.
Figure S3. Generation and activation of monocyte-derived macrophages. Human monocyte-derived macrophage (MΦ) phenotype was confirmed by A) flow cytometry (CD14+CD68+CD80+CD86+) and B) their ability to release solTNF and VEGF in response to LPS + IFNγ activation.

## Slide 6
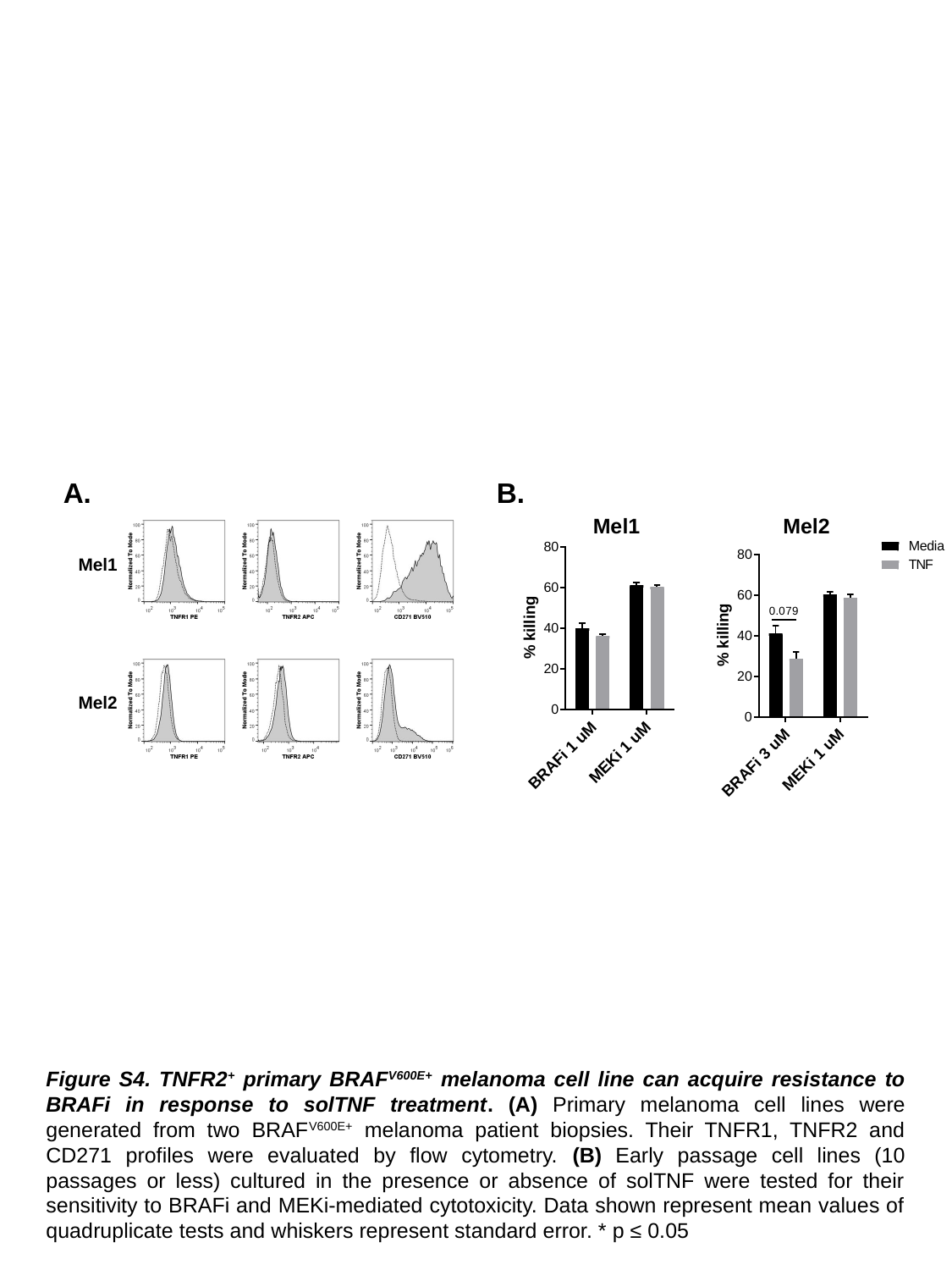

A.
B.
Mel1
Mel2
Mel2
Mel1
Figure S4. TNFR2+ primary BRAFV600E+ melanoma cell line can acquire resistance to BRAFi in response to solTNF treatment. (A) Primary melanoma cell lines were generated from two BRAFV600E+ melanoma patient biopsies. Their TNFR1, TNFR2 and CD271 profiles were evaluated by flow cytometry. (B) Early passage cell lines (10 passages or less) cultured in the presence or absence of solTNF were tested for their sensitivity to BRAFi and MEKi-mediated cytotoxicity. Data shown represent mean values of quadruplicate tests and whiskers represent standard error. * p ≤ 0.05

## Slide 7
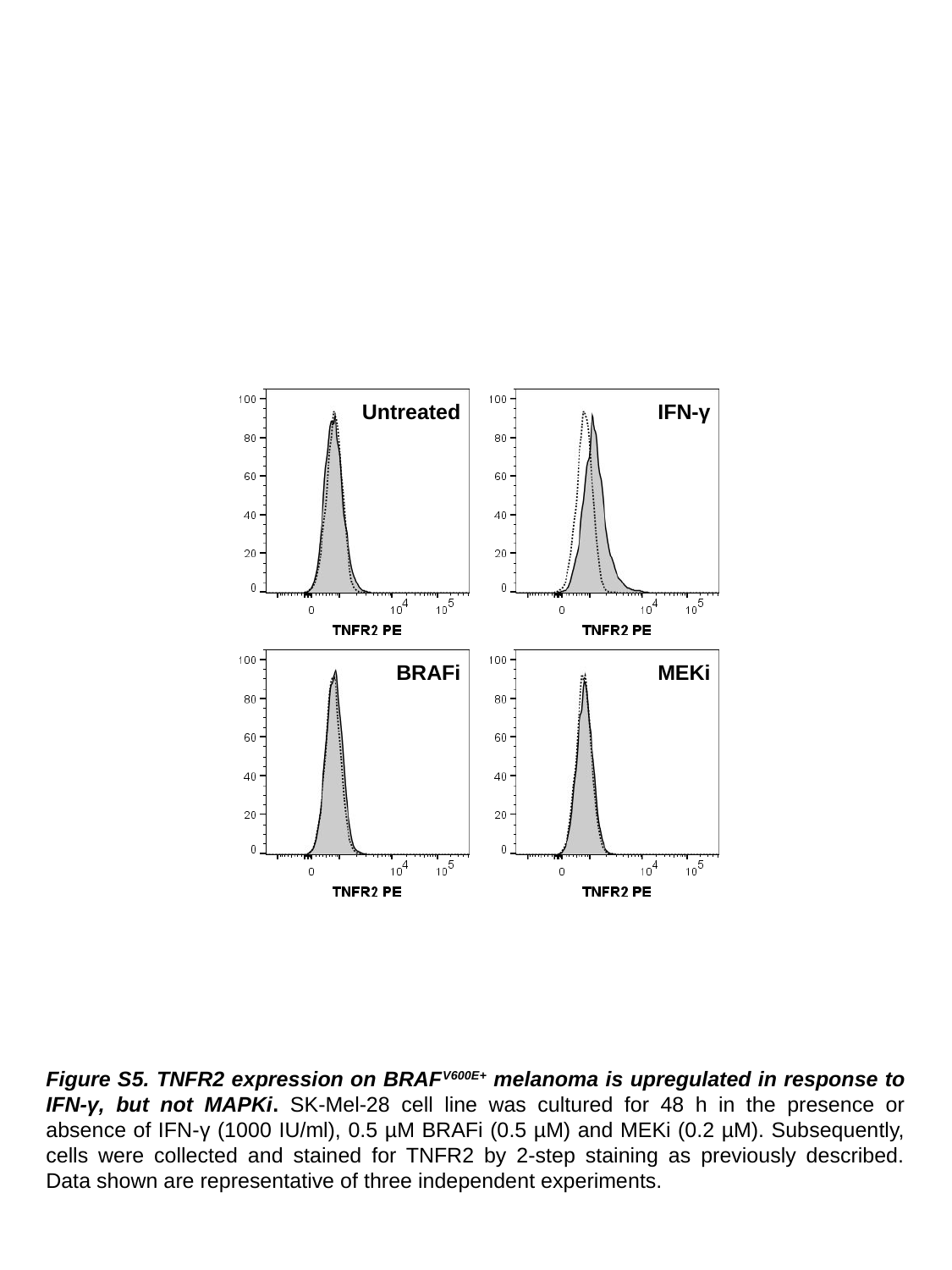

IFN-γ
Untreated
MEKi
BRAFi
Figure S5. TNFR2 expression on BRAFV600E+ melanoma is upregulated in response to IFN-γ, but not MAPKi. SK-Mel-28 cell line was cultured for 48 h in the presence or absence of IFN-γ (1000 IU/ml), 0.5 µM BRAFi (0.5 µM) and MEKi (0.2 µM). Subsequently, cells were collected and stained for TNFR2 by 2-step staining as previously described. Data shown are representative of three independent experiments.
